# Career regret: an analysis of physician assistants

**DOI:** 10.15694/mep.2019.000037.1

**Published:** 2019-03-04

**Authors:** Talia Sierra, Melanie Domenech Rodriguez, Jennifer Forbes

**Affiliations:** 1 Idaho State University; 2 Utah State University

**Keywords:** career regret, career satisfaction, burnout, physician assistant

## Abstract

This article was migrated. The article was marked as recommended.

**Purpose:** The purpose of this study was to identify life and career variables that differ between physician assistants (PAs) with and without career regret. The information in this article may be useful to PAs and PA students in their search for a professional environment that is associated with a lower risk of career regret.

**Methods:** A survey was emailed to 5,000 PAs nationally. Aspects of their life and careers were compared between those with career regret and those without.

**Results**: 26.9% of respondents indicated career regret. Differences were found between PAs with and without regret on the degree of schedule control, hours worked per week, salary, work-life balance, perceived burnout, career satisfaction, advice to others considering the career, and work stability. Other elements that were analyzed were not statistically significant between groups.

**Conclusion:** Differences in work and life aspects were found when comparing PAs with and without regret. Current and future PAs may use the information from this study to help create or seek professional environments that will be less likely to lead to burnout and career regret. Employers can also utilize the information from this research study to develop or maintain work environments that protect against burnout.

## Introduction

The physician shortage in the United States is a longstanding and well-known healthcare dilemma with future projections painting a bleak picture ( Pentecost, 2017; *Association of American Medical Colleges*, 2018). The need for healthcare providers is greater than ever. The physician assistant (PA) profession can help address this issue, particularly the primary care physician shortage ( Cawley and Hooker, 2010; Hooker and Everett, 2012). The first class of PAs graduated in 1967 (‘AAPA’, no date)and over the last fifty years the PA profession has gained acceptance in medicine and the number of practicing PAs continues to rise ( Rittle, 2018).

Career regret among physicians has been studied extensively ( Lemkau, Rafferty and Gordon, 1994; Halbesleben and Rathert, 2008; Adams, 2012; *Beckers Hospital Review*, 2014; Drummond, 2015; Lowes, 2015; Shanafelt, Dyrbye, *et al.*, 2016; Shanafelt, Mungo, *et al.*, 2016; West, Dyrbye and Shanafelt, 2018); however there is limited research specifically pertaining to PAs with regard to career regret. Although studies have reported high rates of career satisfaction among PAs ( Labarbera, 2004; ‘Job Satisfaction’, 2017; *Beckers Hospital Review*, 2017) being merely satisfied with one’s profession does not necessarily indicate they would pick the same career again. PAs report high satisfaction rates, while simultaneously reporting high burnout rates ( Sierra, Forbes and Nelson, 2018).

Career regret is defined as “a negative emotion generated by comparing between actual and expected (better) outcomes” ( Li, Hou and Jia, 2015) while burnout is “a constellation of symptoms experienced by workers as they respond to the demands of their work with inadequate resources to buffer stress” ( Lemkau, Rafferty and Gordon, 1994). While burnout and career regret are separate constructs, burnout does lead to a higher likelihood of dissatisfaction and career choice regret ( Dyrbye *et al.*, 2018). The effect of burnout on patient care has been well documented ( Halbesleben and Rathert, 2008; Drummond, 2015; Hooker, Kuilman and Everett, 2015; Peckham, 2015). It has been linked to higher medical errors, poorer quality outcomes, decreased patient satisfaction and compliance, and can lead to substance abuse and mental health problems ( Drummond, 2015; West, Dyrbye and Shanafelt, 2018). Provider turnover resulting from burnout is also costly, making an investment in prevention worthwhile( Waldman *et al.*, 2010; AAPA Research Department, 2018).

Control over one’s practice environment was cited as the most important contributor to practice satisfaction in both physicians ( Peckham, 2015) and PAs ( Warner, Maio and Hudmon, 2013). Elements that contribute to career regret among physicians include declining incomes, excessive paperwork, electronic health records and working too many hours ( Adams, 2012; Peckham, 2015). Other factors considered in the literature that may lead to career regret include student loan debt, marital status and gender ( Peckham, 2015; Phillips, 2016).

The purpose of this study is to identify life and career variables that contribute to career regret in PAs. Having an awareness and understanding of these elements early in a medical career will help to facilitate and build lifestyle habits that will prevent career regret. It also gives clinicians a tool to identify which items are essential to negotiate for when entering into a new contract in order to set themselves up for a long and successful career and put them in a position to deliver high-quality care.

## Methods

The authors developed a 24-item survey for the study that requested demographic data and information about the participants’ career and life as a PA. The authors asked about perceived level of burnout, sustainability of work/ life balance, control of schedule, satisfaction regarding their PA career, if they would choose to go to PA school again, how they would advise someone considering PA school, frequency of job changes, salary, number of jobs held as a PA, and number of years working as a PA. The research was reviewed by the Institutional Review Board at Idaho State University (IRB-FY2017-131) and was determined to be exempt ( Internal Review Board, 2017). Data were collected anonymously.

The American Academy of PAs (AAPA) sent a complete list of members (20,000+) with permission to select 5,000 for use. Once the list was received, 1,250 PAs were randomly selected from each internal medicine and family medicine with an additional 2,500 randomly selected from 18 specialty care areas. We e-mailed 5,000 PAs as agreed upon with the AAPA. The 5,000 selected PAs were emailed invitations to participate via Qualtrics Online Survey Software along with information regarding the study and participation. Participants indicated their consent by proceeding to the survey. Eight hundred thirty four participants responded to the survey for a response rate of 16.7%.

Respondents endorsing career regret were determined by correlating responses to one item on the survey, “if you could choose again, would you choose to become a PA or choose a different career path?” with The Decision Regret Scale, a previously validated instrument in measuring decision regret ( Brehaut *et al.*, 2003). This item demonstrated concurrent validity, *r*(790)= .74, *p* <.001,and reliability, cronbach alpha = .89, with the validated Decision Regret Scale.

Statistical analysis focused on group comparisons. Independent samples *t* tests were performed on continuous data and chi square analysis were performed on categorical data.

## Results/Analysis

### Demographic characteristics

Of the respondents, 216 (26.9%) indicated career regret. See Table 1 for demographic data between the regret and no-regret groups. Overall, PAs were 25 to 78 years old ( *M* = 42.40, *SD* = 12.19). The mean age for those with and without regret was 42.91 and 41.91 years. There was no significant difference in mean age between those that reported career regret and those that did not, *t*(625) = -.93, *p* = .353. The respondent PAs were married ( *n* = 617, 74.0%), single ( *n* = 154, 18.5%), divorced ( *n* = 47, 5.6%), and widowed ( *n* = 8, 1.0%). There was no significance in marital status between those with career regret and those without, χ ^2^(3) = 3.55, *p* = .315. Many PAs did not have children ( *n* = 322, 38.6%) and there were no statistical differences between the groups in the number of children they had, χ ^2^(5) = 5.63, *p* = .343. Those with no regret had a mean of 2.33 children ( *SD* = 1.31) and those with regret reported a mean of 2.22 children ( *SD* = 1.21).

Respondent PAs were predominantly women ( *n* = 615, 73.7%). There were 209 men (25.1%) and 10 participants (1.2%) did not provide a response. There were no significant gender differences between the career regret and no-regret groups, χ ^2^(2) = 3.89, *p* = .143. Participants were primarily White American ( *n* = 704, 84.4%), followed by Asian American ( *n* = 30, 3.6%), Black American ( *n* = 23, 2.8%), Latino ( *n* = 21, 2.5%), Middle Eastern or North African ( *n* = 6, 0.7%), Native Hawaiian or Other Pacific Islander ( *n* = 3, 0.4%). Ten participants (1.2%) reported “other race/ethnicity”. Because the cells were small for comparison purposes, ethnic minorities were collapsed into one group and compared with White Americans. There were no significant differences in the proportion of ethnic minorities that would attend PA school again or not compared to White Americans, χ ^2^(1) = 1.147, *p* = .284.

Participants reported on the question “How many jobs do you currently have in which you are employed as a PA?” Response options were 0 ( *not currently employed as a PA*), 1, 2, 3, or 4 ( *4 or more*). 96.5% of respondents were currently employed as PAs ( *n* = 786) and 77.2% ( *n* = 644) had one job as a PA. There were no differences in career regret across job categories, χ ^2^(4) = 2.338, *p* = .674. Respondents were PAs in all 50 states. The highest representation of PAs was from Texas ( *n* = 62, 7.4%) and New York ( *n* = 59, 7.2%).

**Table 1.** Demographic Data

	No-Regret Group *n*	Regret Group *n*	Overall Sample *n*
Gender			
Male	157	48	205
Female	430	165	595
Prefer not to answer	1	2	3
Total	588	215	803
Ethnicity			
Caucasian	505	177	682
Asian	20	10	30
Hispanic, Latino or Spanish Origin	16	4	20
Black or African American	15	7	22
Other	13	8	21
Prefer not to answer	19	10	29
Total	588	216	804
Relationship Status			
Married	441	159	600
Single	109	41	150
Divorced	30	14	44
Widowed	8	0	8
Total	588	214	802
Number of Children			
0	235	87	322
1	75	31	106
2	168	71	239
3	77	17	94
4	25	7	32
5 or more	8	2	10
Total	588	215	803
Number of Jobs Currently Held			
0	13	5	18
1	468	176	644
2	84	27	111
3	14	7	21
4 or more	9	1	10
Total	588	216	804

### Work-related variables

*Perceived control over schedule.* Participants indicated their degree of perceived control over their schedules by responding to one survey item, “To what degree do you have control or say over your schedule?” A continuous scale ranging from 1 ( *no control*) to 5 ( *total control*) was used. The difference between the regret and no-regret groups was statistically significant. The no-regret group reported higher control over their schedule ( *M* = 3.45, *SD* = 1.03) than the regret group ( *M* = 2.86, *SD* = 1.15), *t*(799) = 6.68, *p* < .001.

*Work hours.* Respondent PAs reported on the number of hours they worked per week by responding to one survey item, “On average how many hours per week do you work as a PA?” Participants could choose 20 or less, 20 to 30, 30 to 40, 40 to 50, 50 to 60, 60 or more. A chi square analysis reflects a significant difference between groups, χ ^2^(5) = 13.10, *p* = .023. The significant differences were for those that worked 20 - 30 hours and those that worked 60 or more hours. Both of those groups had higher proportions of PAs who would chose a different career (see Table 2).

**Table 2.** Number of hours worked per week in those with career regret and those without (%)

	Regret Group	No-Regret Group
20 or less	4.62	4.25
20-30 hours	11.57	6.80
30-40 hours	26.85	29.76
40-50 hours	40.74	45.75
50-60 hours	10.19	11.22
60 or more hours	6.01	2.21

*Years as a PA.* Participants were asked “How many years have you been practicing as a PA?” Respondents could select one of 6 categories: less than 1 year, 1-2 years, 2-5 years, 5-10 years, 10-15 years, and more than 15 years. There was no statistical significance in the regret and no-regret groups based on length of practice, χ ^2^(5) = 7.38, *p* = 0.194. See Table 3 for distribution between the groups.

**Table 3.** Years of Practice in those with career regret and those without (%)

	Regret Group	No-Regret Group
Less than 1 year	3.72	5.95
1-2 years	5.58	7.99
2-5 years	15.81	18.71
5-10 years	16.28	19.05
10-15 years	20.93	17.35
More than 15 years	37.67	30.95

*Salary.* PAs were asked “If you are employed full-time, what is your yearly gross salary after all pay sources have been included (base pay, bonuses, incentives, call pay, etc.)?” Participants responded across 8 categories that ranged from (1) *less than $70,000* to (8) *more than $140,000.* A chi square analysis revealed statistically significant differences between the regret and no-regret groups χ ^2^(7) = 18.41, *p* = .010. A z-test to compare column proportions and using a Bonferroni correction, revealed significant differences in regret in the *70,001 - 80,000*, *80,001 - 90,000,* and *90,001 - 100,000* groups. The *$70,001 - $80,000* and the *$90,001 - $100,000* groups had a higher proportion of PAs experiencing regret. Specifically, 6.4% of the regret sample fell in the *$70,001 - $80,000* group compared to 3.0% of the no-regret sample, and 25.5% of the regret group reported earning *90,001 - 100,000* compared to 17.4% of the no-regret group. Curiously, the relationship was reversed with a higher proportion of the no-regret group reporting earning $80,001-$90,000 at 10.4% than the regret group at 5.3%.

*Sustainable work-life balance.* Sustainable work-life balance was addressed using one survey item, “To what extent do you agree your career as a PA has provided you with a suitable work-life balance?” on a scale from 1 ( *strongly agree*) to 5 ( *strongly disagree*). Mean differences between groups were highly significant, *t*(309.891) = -10.06, *p* < .001. The no-regret group had a higher agreement ( *M* = 1.69, *SD* = 0.90) with the statement than the regret group ( *M* = 2.58, *SD* = 1.18).

*Work stability.* PAs were asked “How often have you left a position or changed specialties due to the following: Dissatisfaction with the specialty, workload was too heavy, unpleasant/hostile work environment, and poor work/life balance.” Participants provided a number between 1 and 10. In regards to dissatisfaction with the specialty, there were no statistically significant mean differences between those with and without career regret, *t*(162.961) = -1.69, *p* = .094. The same was true for heavy workload, *t*(241) = -1.24, *p* = .215. Mean differences between groups were significant for unpleasant work environment, *t*(156.738) = -2.23, *p* = .027, where those without career regret changed jobs less frequently ( *M* = 1.32, *SD* = 1.31) than those with regret ( *M* = 1.53, *SD* = 1.19). Mean differences between groups were also significant for poor work-life balance, *t*(168.246) = -2.87, *p* = .005. The no-regret group had lower means ( *M* = 1.30, *SD* = 0.82) than the regret group ( *M* = 1.71, *SD* = 1.48).

### Life and Satisfaction Variables

*Advice to others.* When asked “If you could choose again, would you choose to become a PA or choose a different career path?” those PAs that expressed regret were likely to give different advice that those without regret, χ ^2^(3) = 347.13, *p* < .001. Those with regret were more likely to recommend medical school, a different health care profession, or a different field altogether. Those without regret were more likely to recommend attending PA school.

*Student loan debt.* Participants responded to the item: “Please indicate the amount of student loan debt you had upon graduating from PA school”. There were no significant differences in career regret across participants in the eight debt categories that ranged from *less than 20,000*to *more than 190,000*, χ ^2^(7) = 11.45, *p* = .120.

*Burnout.* Burnout was measured with a one item scale, “Please indicate your current perceived level of burnout”. Respondents chose a value between 1 ( *no burnout*) and 5 ( *ready to quit*). Mean difference in burnout ratings between those who would attend PA school again ( *M* = 2.27, *SD* = 1.03) and those that would not ( *M* = 3.40, *SD* = 1.07) were highly significant, *t*(738) = -13.286, *p* < .001 indicating that those who would not attend PA school again had higher self-rated burnout than those that would.

Statistical significance was found with higher rates of burnout as years of practice increased. A one-way analysis of variance revealed significant mean differences between groups on burnout ratings, F(9, 744) = 2.858, p = .015. The differences were most notable for the groups of PAs that had be practicing less than one year (M = 2.12) when compared with those who had been practicing 10 - 15 years (M = 2.72) or 15 or more years (M = 2.71), with Tukey posthoc significance of .042 and .27 respectively. Scores correlating with lower burnout decrease with more years of practice (scores of 1 and 2), while the higher burnout scores trend upwards (scores of 4 and 5).

*Satisfaction with career choice.* Respondents’ satisfaction with their career choice was measured with one survey item, “How satisfied are you with your career choice as a PA?” Respondents selected an answer between 1 ( *extremely satisfied*) and 5 ( *extremely dissatisfied*). There was a highly statistically significant difference between those that would attend PA school again ( *M* = 1.32, *SD* = 0.534) and those that would not ( *M* = 2.59, *SD* = 1.12), *t*(249.613) = -16.11, *p* < .001 with those demonstrating career regret reporting lower career satisfaction than those without career regret.

Worthwhile sacrifice. When asked “Do you feel your profession was worth the sacrifices made?” most participants ( *n* = 738, 88.5%) responded yeseven though a sizeable number of those that responded yes would chose a different career path if given the option to choose again ( *n* = 151, 20.5%). Of the 67 participants that did not feel the profession was worth the sacrifices, only 6 reported that they would attend PA school again; most ( *n* = 61, 91.0%) regretted becoming a PA. The differences between the regret and no-regret group was highly statistically significant, χ2(1) = 156.41, *p* < .001.

## Discussion

The most recent national data on certified PAs from the National Commission on Certification of Physician Assistants (NCCPA) reports that 68.2% of certified PAs identify as female ( *2017 Statistical Profile of Certified Physician Assistants*, 2018). Our respondent pool was similar with 73.7% identifying as such. Additionally, the NCCPA categorizes age groups, with the 30-39 age group being the highest endorsed at 38.2% followed by the 40-49 age group at 23.5% ( *2017 Statistical Profile of Certified Physician Assistants*, 2018). This is consistent with our findings of 42.4 years, indicating that our sample pool is a good representation of PAs practicing nationally.

Distinct predictors of career regret were identified in this national sample of PAs. Specific indicators were degree of control over schedule, hours worked per week, salary, work-life balance, perceived burnout, career satisfaction, and work stability. Conversely, elements that have been implicated in career regret, such as higher student loan debt, marital status and gender were not statistically significant in our analyses ( Peckham, 2015; Phillips, 2016).Additionally, many respondents who would not attend PA school again indicated they still felt the profession was worth the sacrifices made, an unexpected finding.

Our findings with regards to work hours indicate that those working 20-30 hours per week and those working 60+ hours per week had higher rates of respondents in the career regret group. Our findings are consistent with research that shows that overworking leads to burnout ( Carmichael, 2015). Conversely reduced hours as a PA may be a consequence of burnout ( Shanafelt, Mungo, *et al.*, 2016). The cross-sectional design does not allow for an examination of causal relationships. Future research may seek to replicate these findings with PAs.

We also found that those with lower salaries ($70,001-80,000) had higher proportions of career regret. It appears that the relationship between income and regret is complex at the lower levels and is an area for future research with focus on more nuanced data collection such as motivations for pursuing a career as a PA or contextual variables such as the willingness to earn less for a more satisfying position.

Those in the career regret group reported less control over their schedule than those without regret. This supports prior research showing that lack of control over one’s schedule is one of the largest predictors of career regret ( Warner, Maio and Hudmon, 2013; Peckham, 2015). Employers can help prevent burnout and career regret by accommodating individual schedules and needs. Additionally, those in the regret group cited leaving positions more frequently due to an unpleasant work environment and poor work-life balance. There was no difference between the groups in leaving positions due to dissatisfaction with the specialty or heavy workload. This may indicate that unpleasant work environment and poor work-life balance are elements that specifically contribute to career regret.

Our research methods could be improved in future research. Our study had a low response rate, key variables were measured with one question and those questions had categorical rather than continuous response options. These issues limit our generalizability and precision; however, the survey was constructed by PAs with the knowledge that busy schedules and excess of email requests for participation in various professional activities could reduce response rates even further. In addition to examining work contexts, future research may focus on clinical intervention. Mindfulness is one of various interventions that appears to help reduce burnout among providers ( Peckham, 2015; Karr, 2018).

## Conclusion

Information from this research study may be useful to PAs and PA students to combat the effects of burnout and career regret. Work environments that allow for schedule flexibility, control of work schedule, and total 30 and 60 hours of work per week were shown to be associated with lower levels of burnout and career regret. Additionally, the importance of work-life balance is apparent. PAs should form habits that create boundaries between their personal and professional lives in order to ensure their personal and leisure time is protected from work distractions. Establishing these habits early in one’s career may help to combat career regret. PAs should also seek employers who support or encourage personal time away from work.

## Take Home Messages

• It is important for PAs to protect themselves from career regret and to seek supportive work environments that help to prevent career regret.
• Schedule control, work-life balance, salary, perceived burnout, work stability, work-life balance, career satisfaction and hours worked showed significant differences between those with career regret and those without.
• The significant factors should be considered when negotiating a contract.
• PAs should develop and form boundaries between work and personal life in order to maintain work-life balance.

## Notes On Contributors

Talia Sierra, MPAS, PA-C, earned her MPAS in 2008 from Idaho State University where she is currently employed as an Assistant Professor. Her clinical experience includes family practice, psychiatry, and urgent care.

Melanie M. Domenech Rodriguez, Ph.D., is a Professor of Psychology at Utah State University. Her clinical and research expertise is in the effective implementation and cultural adaptation of evidence-based interventions to improve parenting practices. She is a Fellow of the American Psychological Association and the Association for Psychological Science.

Jennifer Forbes, MHS, PA-C, earned her MHS in 2001 from University of South Alabama. She is currently employed at the Idaho State University PA program. Her clinical experience includes family medicine, pediatrics, pediatric and adult orthopedics.
