# Supplementary material for: Career regret: an analysis of physician assistants
Source: MedEdPublish (2016). 2019 Mar 4;8:37. [Version 1] doi: 10.15694/mep.2019.000037.1 (PMC10712601; doi:10.15694/mep.2019.000037.1)
Supplement: Peer review history

# Reviewer report: Reviewer response for version 1

**DOI:** 10.21956/mep.19763.r29672

**Published:** 2019-03-17

**Authors:** Rille Pihlak

**Affiliations:** 1 The University of Manchester

This review has been migrated. The reviewer awarded 4 stars out of 5 This is a very interesting piece of work that shows clearly that PA-s have similar problems with their work as other medical professionals. I think it is very important to use the results of this study to improve the working conditions of PA-s.In terms of the paper itself- I noticed that under the "advice for others" sections- there might have been a wrong question added or did they just asked the same question about regret.Also, I would have to agree that validated questionnaires especially for burnout, would have given more depth to the research and showed more objective results.In general- I do think this research is very useful and gives quite clear guidance on what could be done differently in order to keep more PA-s and so they would not regret their career choices.

Reviewer Expertise:

NA

No decision status is available

# Reviewer report: Reviewer response for version 1

**DOI:** 10.21956/mep.19763.r29674

**Published:** 2019-03-11

**Authors:** Ariela Marshall

**Affiliations:** 1 Mayo Clinic

This review has been migrated. The reviewer awarded 4 stars out of 5 Thank you for the invitation to review the paper. It is very well done and explores multiple domains of career satisfaction and work-life integration which may have an influence on the presence or absence of career regret. The authors have done a nice job exploring the association of these issues (salary, hours worked, burnout, etc) on career regret. In most cases, the Likert scales used to make these assessments seem very appropriate. The only thing I would have liked to see done differently was to actually use a formal burnout tool (Maslach burnout inventory, etc) to assess burnout rather than simply asking participants whether they self-perceive burnout. I believe there may also be validated instruments for measuring job satisfaction (though this is not my area of expertise). However, while use of validated instruments may add to the validity of the observations, this is not to say that the paper does not raise some very good points. The discussion was well done. Overall I congratulate the authors on a fascinating and meaningful study.

Reviewer Expertise:

NA

No decision status is available

# Reviewer report: Reviewer response for version 1

**DOI:** 10.21956/mep.19763.r29671

**Published:** 2019-03-09

**Authors:** Paolo Colet

**Affiliations:** 1 Nazarbayev University

This review has been migrated. The reviewer awarded 4 stars out of 5 Overall the different section of the article beginning from the introduction down to the take-home message was well written. For the methods, I wonder if there was any validity or reliability testing done for the questionnaire or pretesting of the survey instrument, as this would give more strength to the study.

Reviewer Expertise:

NA

No decision status is available

# Reviewer report: Reviewer response for version 1

**DOI:** 10.21956/mep.19763.r29673

**Published:** 2019-03-04

**Authors:** Trevor Gibbs

**Affiliations:** 1 AMEE

This review has been migrated. The reviewer awarded 4 stars out of 5 Thank you for asking m to review this paper. I enjoyed reading it and was pleased to see a paper written about PAs and job satisfaction.Although it was related to the US situation, I think some of the findings could transfer to other countries such as the UK and as such I feel that this paper needs to be read by all those involved with such a scheme

Reviewer Expertise:

NA

No decision status is available
